# miR-150 enhances apoptotic and anti-tumor effects of paclitaxel in paclitaxel-resistant ovarian cancer cells by targeting Notch3

**DOI:** 10.18632/oncotarget.20348

**Published:** 2017-08-18

**Authors:** Tae Hoen Kim, Ju-Yeon Jeong, Ju-Yeon Park, Se-Wha Kim, Jin Hyung Heo, Haeyoun Kang, Gwangil Kim, Hee Jung An

**Affiliations:** ^1^ Department of Pathology, CHA Bundang Medical Center, CHA University, Gyeonggi-do, Korea; ^2^ Institute for Clinical Research, CHA Bundang Medical Center, CHA University, Gyeonggi-do, Korea

**Keywords:** ovarian cancer, miR-150, Notch3, chemoresistance, sensitization

## Abstract

Tumor recurrence by obtaining chemoresistance is a major obstacle to treating ovarian cancer. By TargetScan database and a luciferase reporter assay, we identified miR-150 directly targets *Notch3*, which is a key oncogene in ovarian cancer. We, therefore, investigated the role of miR-150 in ovarian cancer cells, and the usefulness of miR-150 as a therapeutic target in chemoresistant ovarian cancer, through examining miR-150 expression by qRT-PCR in ovarian cancer cell lines and tissues, and assessing the gain-of-function effect by WST, colony forming, TUNEL, wound healing and angiogenesis assays. Western blotting was performed to evaluate its downstream targets.

The miR-150 expression was significantly downregulated in ovarian cancers. Treatment with pre-miR-150 significantly inhibited cancer cell proliferation, and induced apoptosis in PTX (paclitaxel) -resistant SKpac cells, which was not seen by PTX only treatment. On spheroid forming assay, an additional pre-miR-150 treatment with PTX decreased cancer stem cell activation in PTX-resistant SKpac cells. An experimental upregulation of miR-150 also decreased cancer cell migration and angiogenesis in SKpac cells. The Notch3 downstream proteins(NICD3 and HEY2), and cell cycle-related proteins (cyclinD3, pS6, and NF-kB), and apoptosis-related proteins (BCL-2 and BCL-W) were significantly downregulated by pre-miR-150 transfection.

Taken together, miR-150 is related with PTX-resistance in ovarian cancer, and treatment with pre-miR-150 resensitizes cancer cells to PTX. Therefore, it may be a promising treatment strategy in chemoresistant and recurrent ovarian cancer.

## INTRODUCTION

Ovarian cancer is the most lethal gynecologic malignancy worldwide. Most patients with ovarian cancer are usually diagnosed at the more advanced stages, and are primarily treated with cytoreductive surgery followed by chemotherapy, composed of platinum and taxane. Despite high initial response rates, as many as 80% of ovarian cancer patients experience recurrence after the first-line chemotherapy, and the five-year survival rate is only approximately 40% in women with advanced ovarian cancer [1, 2]. Resistance to chemotherapy is a major obstacle to a long-term remission, and thus, effective strategies to overcome drug resistance would have significant clinical impact. Understanding of the mechanisms of chemoresistance remains a challenge in ovarian cancer research, and novel treatment strategies to resensitize the resistant ovarian cancers are needed.

Recent studies, including TCGA data, have disclosed that the alteration of Notch pathway is found in 22% of analyzed cancers and is one of the most altered pathways in serous type ovarian carcinoma [3]. Interestingly, we now have data showing that the Notch signaling pathway, Notch3 in particular, plays a key role in cancer stem cell (CSC) maintenance and paclitaxel(PTX)-resistance [4, 5]. In addition, Notch3 RNA transcript and protein are highly expressed in ovarian carcinomas [6] and its elevated expression correlates with resistance to chemotherapy and decreased survival. Moreover, knockdown of Notch3 sensitizes OVCAR3 cells to carboplatin [7]. McAuliffe *et al*. [8] strongly suggest that the Notch3 signaling pathway is important in CSCs maintenance and tumor resistance. Therefore, Notch3 is critical to the regulation of ovarian CSCs and tumor resistance and CSCs are important targets for overcoming chemoresistance. Given that Notch3 signaling supports CSC activity in ovarian cancer, inhibition of the Notch3 signaling could selectively target tumor-initiating populations and act to restore chemosensitivity. This leads to the hypothesis that Notch3 pathway inhibition in ovarian cancer could produce the most robust effects in a PTX-resistant tumor. We have previously demonstrated that Notch3-specific inhibition by Notch3 specific siRNA and γ-secretase inhibitor (GSI) sensitizes PTX-resistant ovarian cancer cells to paclitaxel treatment [5].

MicroRNAs (miRNAs) are a class of small endogenous noncoding RNAs (18 - 22 nucleotides) that elicit their regulatory effects in post-transcriptional regulation of genes by binding to the 3′-untranslated region (3′-UTR) of target messenger RNA (mRNA), resulting in mRNA degradation and/or translational repression [9]. It is well known that deregulated expression of specific miRNAs is associated with various diseases, including solid and hematopoietic tumors. Recent studies have suggested that altered expression of specific miRNAs plays important roles in the regulation of drug resistance in ovarian cancer [10, 11]. Additionally, miRNAs can be used to sensitize tumors to chemotherapy. Therefore, we hypothesized that the inhibition of Notch3 using modulation of miRNAs in PTX-resistant ovarian cancer cells may result in sensitizing the PTX resistant ovarian cancer cells, and inducing inhibition of cellular proliferation and cancer cell death.

Searching by TargetScan, we identified several miRNAs including miR-150, miR-146 and miR-4510, which potentially targets Notch3 mRNA. After confirmation by the promoter assay, we finally chose miR-150 as a predictive miRNA targeting Notch3, and we focused on miR-150 for further study. MiR-150 reportedly has essential regulatory roles in both normal and malignant hematopoietic cells and also has a great potential as a therapeutic target in various types of hematological malignancies [12]. Emerging evidence suggests that miR-150 is downregulated in various types of lymphomas such as mantle cell lymphoma (MCL), conjunctival mucosa-associated lymphoid tissue (MALT) lymphoma, Burkitt lymphoma (BL) and NK/T-cell lymphoma [13]. Ghisi *et al*. [14] showed that forced expression of miR-150 reduced Notch3 levels in T cells and resulted in adverse effects on cell proliferation and survival, suggesting that control of the Notch3 pathway through miR-150 may have an important impact on the growth of T cells, even though the underlying molecular mechanisms are still poorly understood.

In the current study, we first identified that miR-150 is significantly downregulated in a subset of high-grade serous carcinomas (HGSC), which is the most common and lethal subset of ovarian carcinomas, and PTX-resistant SKpac cells. We also investigated the impact of miR-150 manipulation on sensitizing PTX-resistant ovarian cancer cells, and assessed the usefulness of miR-150 as a therapeutic target in patients at an advanced stage with recurrence. In addition, the cellular and molecular effects of pre-miR-150 transfection were assessed, thus providing major insights into the role of miR-150 in ovarian carcinogenesis.

## RESULTS

### miR-150 is downregulated in high-grade serous ovarian cancer (HGSC) and PTX-resistant ovarian cancer cells (SKpac cells)

**Figure 1:**
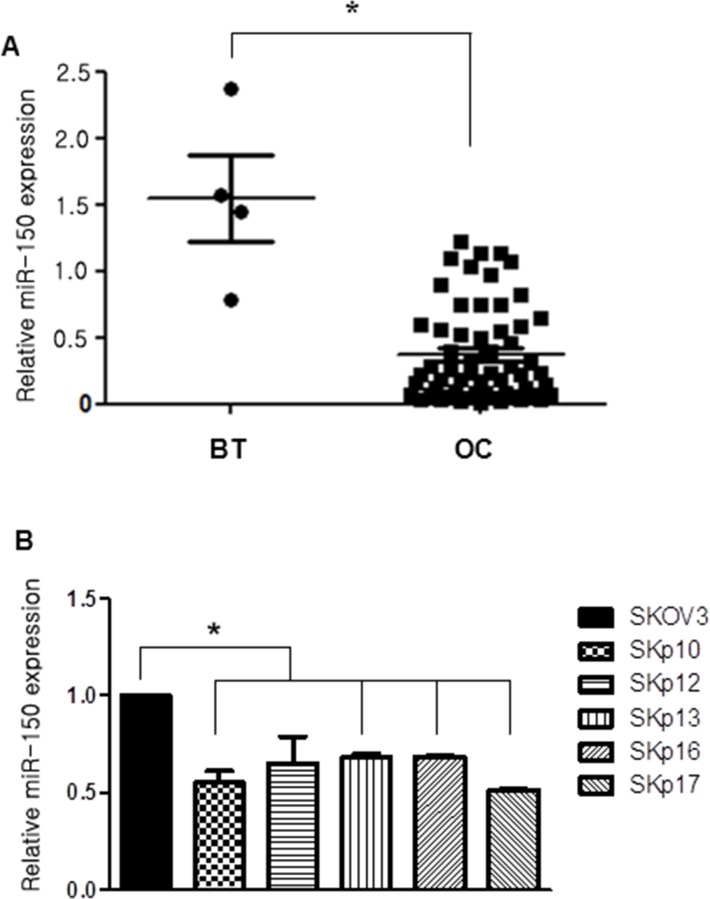
Expression of miR-150 measured by qRT-PCR in ovarian cancer (OC) samples and in chemoresistant ovarian cancer cell lines **(A)** miR-150 expression levels of ovarian high-grade serous carcinoma (OC) cases are significantly downregulated by 0.2-fold than benign ovarian serous tumor (BT) cases (*P*<0.05). **(B)** The relative expression levels of miR-150 in parental SKOV3 ovarian cancer cells versus PTX-resistant Skpac cells. The expression level of miR-150 in PTX-resistant Skpac cells (Skpac-10,−12,−13,−16,−17) were significantly downregulated by 0.5-0.6-fold compared with that of parental SKOV3 ovarian cancer cells (*P*<0.05).

We investigated the expression of miR-150 by qRT-PCR to compare the relative levels of miR-150 in benign ovarian serous tumor (BT) and HGSC samples (OC), and observed a significant downregulation of miR-150 levels by 0.2-fold in HGSC cases compared with BT cases (*P*<0.001, Figure 1A). To investigate whether endogenous miR-150 levels are associated with chemoresistance, the expression level of miR-150 was quantified using real time qRT-PCR analysis of RNAs isolated from SKOV3 and SKpac cells. As a result, the miR-150 expression was significantly lower in PTX-resistant SKpac cells (SKpac-10,−12,−13,−16, and −17) than in SKOV3 parental cancer cells (*P*<0.05, Figure 1B).

### miR-150 regulates Notch3 expression by targeting the 3′UTR of its mRNA

**Figure 2:**
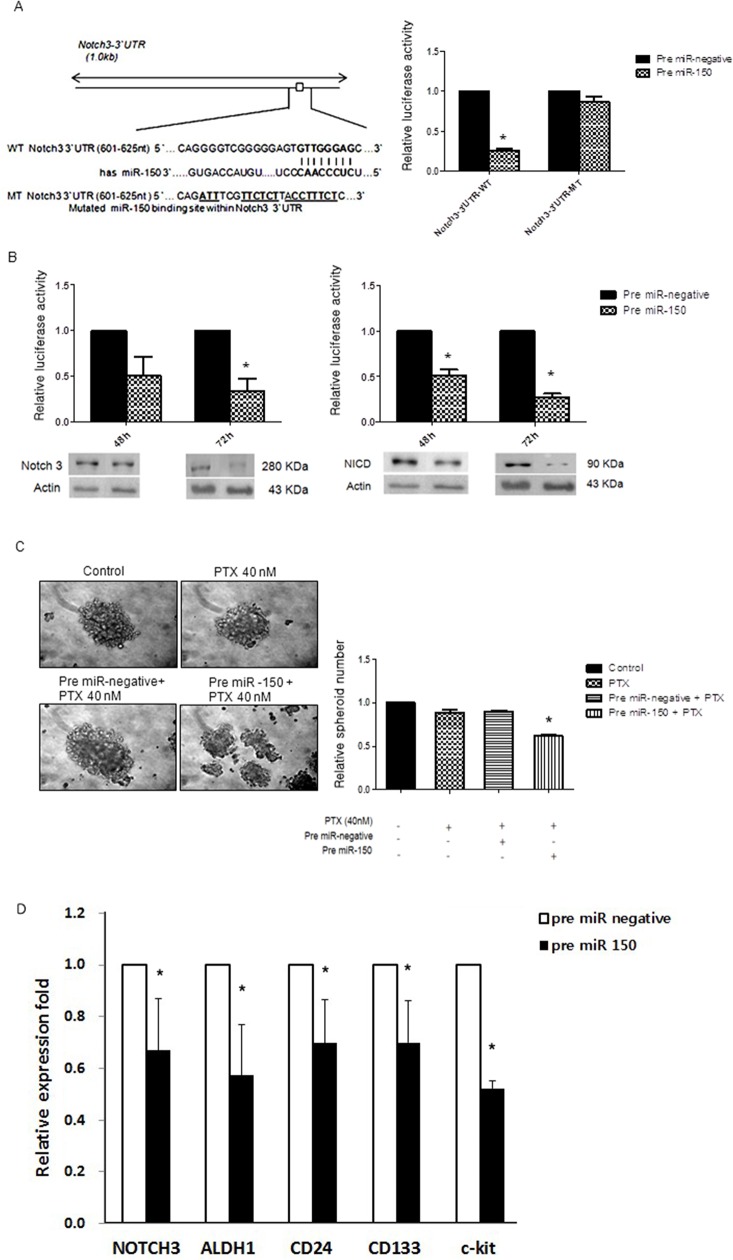
miR-150 directly targets *Notch3* **(A)** Luciferase reporter assay. Left panel: Putative miR-150 target site and mutant in the 3′UTR of *Notch3*. Right panel: Effects of miR-150 on Luciferase activity of the reporter gene bearing wild-type or mutant 3′UTR of *Notch3* in SKpac cells. **(B)** Effects of overexpression of miR-150 on expression of NOTCH3 and NICD3. By Western blotting, reduced expression of Notch3 and NICD3 was shown in SKpac cell lines with pre-miR-150 transfection at 48h and 72 h. **(C)** Spheroid-forming assay. The number and size of spheroids were markedly reduced in SKpac-17 cells transfected with PTX and pre-miR-150 relative to control, PTX only(40nM), or PTX(40nM) + pre-miR-negative siRNA (* *P* < 0.05). **(D)** PTX-resistant SKpac cells (SKpac-12, SKpac-13, and SKpac-17 cells) subjected to pre-miR-150 treatment were analyzed with qRT-PCR to measure mRNA expression of key stem cell markers. The mean mRNA expression levels of NOTCH3, ALDH1, CD24, CD133, and c-Kit were significantly reduced to 0.67-, 0.57-, 0.70-, 0.70-, and 0.51-fold, respectively (*P*=0.046, *P*=0.019, *P*=0.012, *P*=0.031, *P*=0.002, respectively), relative to control (* *P* < 0.05).

A luciferase reporter assay was performed to determine whether miR-150 directly targets Notch3 in SKpac-13, −16, and −17 cell lines. In these experiments, SKOV3 cells were co-transfected with a pGL3 vector containing the firefly luciferase reporter upstreaming of the 3′UTR of *NOTCH3*, a pRL-TKvector expressing Renilla luciferase (as a control), and 50 pmol of pre-miR-150 or a control miRNA. Luciferase activity in the cells transfected with pre-miR-150 was significanlty lower (25±5%) than that in the cells transfected with the control miRNA, whereas pre-miR-150 showed no effect on luciferase activity of reporter contruct containing mutant *NOTCH3* 3′UTR (Figure 2A), indicating that miR-150 directly targets the 3′UTR of *NOTCH3* in SKpac cells. To further confirm that the protein expression of Notch3 and NICD3 is regulated by miR-150, we performed the gain-of-function analysis by forced expression of endogenous miR-150 with pre-miR-150 transfection in SKpac cells, and pre-miR-negative was used as a negative control. MiR-150 was stably overexpressed with a pre-miR-150 transfection in SKpac cells resulting in a 5-fold increase in miR-150 expression (data not shown). Forced expression of miR-150 reduced the levels of both Notch3 and NICD3 to 34%-50% and 27%-52%, respectively, in SKpac-12, and −17 cells (Figure 2B). These findings indicate that miR-150 directly regulates Notch3 and NICD3 expression in SKpac cells.

### miR-150 regulates cancer stem cell activity in SKpac cells

To verify the effect of miR-150 transfection on cancer stem cells (CSCs) activation, we performed spheroid-forming assay. The number of spheroids decreased significantly after PTX + pre-miR-150 transfection, to 0.38-fold relative to PTX alone or PTX + miR-negative treatment (Figure 2C, *P < 0.05). The size of spheroids was markedly reduced on combination treatment of PTX and pre-miR-150 transfected SKpac cells relative to both PTX alone and PTX + miR-negative treatment, indicating that miR-150 induction may inhibit ovarian CSCs activation. Collectively, while PTX alone induced no changes in spheroid formation, but the additional pre-miR-150 treatment with PTX decreased CSC activation in PTX-resistant ovarian cancer cells. To confirm the effect of pre-miR-150 on CSC activation, we also performed real-time RT-PCR for detecting alteration of mRNA of the stemness genes in paclitaxel-resistant SKpac cells. After transfection with pre-miR-150, the mean mRNA expression levels of NOTCH3, ALDH1, CD24, CD133, and c-Kit were significantly reduced to 0.67-, 0.57-, 0.70-, 0.70-, and 0.51-fold, respectively (*P*=0.046, *P*=0.019, *P*=0.012, *P*=0.031, *P*=0.002, respectively) relative to control (Figure 2D), indicating that miR-150 plays an important role in regulating CSCs.

### Upregulation of miR-150 resensitizes chemoresistant ovarian cancer cells to PTX therapy, inhibiting tumor cell growth, and increasing apoptosis in PTX-resistant SKpac cells

**Figure 3:**
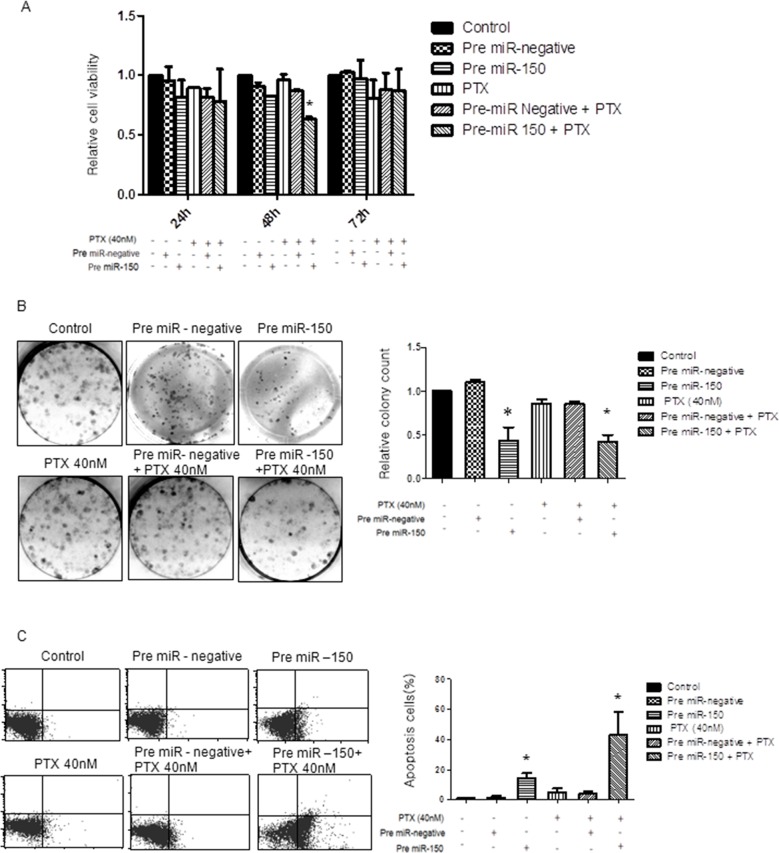
Effect of pre-miR-150 on cancer cell viability, cell proliferation and apoptosis in SKpac-13, 16,17 cells **(A)** WST assay. The cell viability decreased by 34% at 48h in SKpac cells treated with pre-miR-150 and PTX, relative to cells treated with PTX alone. **(B)** Colony forming assay. Both pre-miR-150 transfection only and combination treatment with pre-miR-150 and PTX(40 nM) significantly inhibited colony formation in SKpac cell by 44% and 43%, respectively, relative to cells treated with PTX alone or PTX + pre-miR negative (86%, *P<0.05) **(C)** TUNEL assay. A significant increase of apoptotic cells by 14.4 % and 40.6 %, respectively, is shown in PTX-resistant SKpac cells treated with pre-miR-150 transfection alone and pre-miR-150 transfection with PTX (40 nM), relative to cells treated with PTX alone or PTX + pre-miR negative (3.5% and 4.3%, * *P* < 0.05).

If PTX resistance is causally related to miR-150 downregulation, an artificial augmentation of miR-150 expression levels might modulate PTX sensitivity. To investigate the effect of pre-miR-150 on cancer cell proliferation as a single agent or in combination with PTX chemotherapeutics on SKpac cells (SKpac-12, and −17), we performed cell viability and proliferation test using WST and colony forming assays. In five separate WST assay experiments, SKpac cells were subjected to therapy with either pre-miR-150 alone, PTX alone, pre-miR-150 in combination with PTX, or pre-miR-negative alone. When the cells were treated with pre-miR-150 transfection in the presence of 40 nM PTX, the number of SKpac cells decreased by 34% at 48h, relative to the cells treated with PTX alone. Administration of pre-miR-150 showed decrease tumor cell viability at 48h, however it was not statistically significant. In all analyzed cases, an enhanced anti-tumor activity was found in SKpac cells treated with the combination of pre-miR-150 and PTX, compared with PTX alone and PTX + pre-miR negative (Figure 3A, *P< 0.05).

Next, to further examine the anti-proliferative effect of PTX or pre-miR-150 alone or together on the growth of SKpac cells, colony forming assays were performed. The results revealed that both pre-miR-150 transfection only and combination treatment with pre-miR-150 and PTX(40 nM) significantly inhibited clonal growth of SKpac cells, decreased by 44% and 43%, respectively, relative to the cells treated with PTX alone or PTX + pre-miR negative (86%, *P<0.05) (Figure 3B). Collectively, these findings suggest that miR-150 has an anti-proliferative effect on PTX-resistant SKpac cells either when used alone or in combination with PTX.

To determine the effect of miR-150 on apoptosis, SKpac cells (SKpac-12, 16, 17) were transfected with pre-miR-150 and then analyzed using a TUNEL assay. In Figure 3C, compared with the corresponding control cells, a significant increase in the apoptotic cell population was shown in PTX-resistant SKpac cells treated with pre-miR-150 transfection alone and in combination of pre-miR-150 transfection and PTX (40 nM) increased by 14.4 % and 40.6 %, respectively, relative to cells treated with PTX alone or PTX + pre-miR negative (3.5% and 4.3%, *P<0.05). Taken together, pre-miR-150 transfection resulted in reduced tumor cell proliferation and induction of apoptosis in PTX-resistant ovarian cancer cells by itself and by increasing tumor cell sensitivity to PTX.

### The inhibitory effect of pre-miR-150 on cell migration and angiogenesis in PTX-resistant SKpac cells

**Figure 4:**
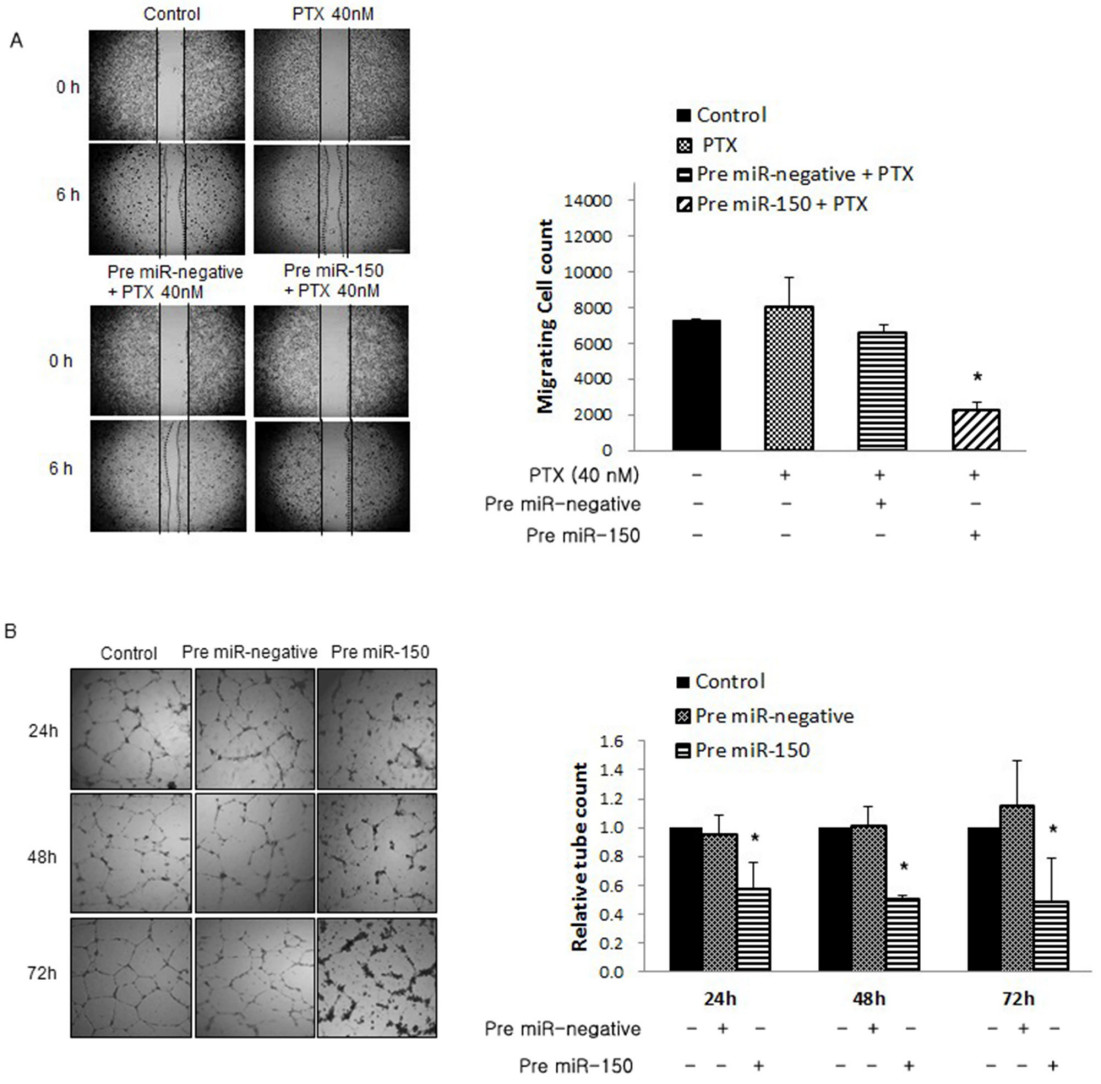
Effects of pre-miR-150 on cell migration and angiogenesis **(A)** Wound healing assays. Migrating cells of pre-miR-150 + PTX treated cells markedly decreased by 28.7 % and 36% respectively, compared with those of PTX alone or PTX + pre-miR negative cells (*P<0.05). **(B)** Tube formation assay. The supernatant from pre-miR-150 transfected cells reduced tube formation by 61.1% at 24h, 50.5% at 48h, and 42.6% at 72h, relative to control HUVECs treated with supernatant from pre-miR-negative cells (*P<0.05).

Cell migration and angiogenesis are important factors that influence tumor progression and metastasis. We examined the effect of pre-miR-150 on cell migration by wound healing assay, and also its effect on angiogenesis by tube formation assay. On wound healing assay, migrating cells of pre-miR-150 + PTX 40nM treated cells were markedly decreased compared with those of PTX alone or PTX + pre-miR negative treated cells (28.7 % and 36% respectively, *P<0.05) (Figure 4A). On tube formation assay, pre-miR-150 transfected cells reduced tube formation by 61.1% at 24h, 50.5% at 48h, and 42.6% at 72h, relative to control HUVECs in the pre-miR-negative cells (Figure 4B, *P<0.05). These results indicate that pre-miR-150 transfection significantly inhibits tumor cell migration and angiogenesis in PTX-resistant ovarian cancer cells, an effect that was not observed when cells were treated with PTX alone or PTX + pre-miR negative.

### miR-150 reduces the expression of Notch3 downstream target proteins and proteins of cell survival, cell cycle, and apoptosis

**Figure 5:**
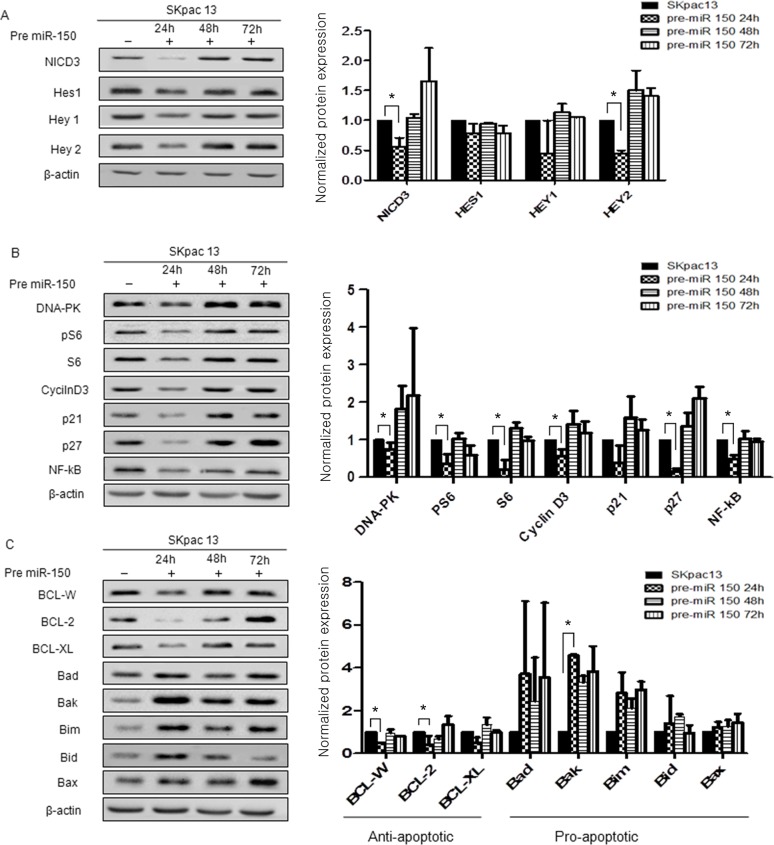
Protein expression in chemoresistant SKpac cells after pre-miR-150 transfection Protein bands were quantitated by densitometric analysis and normalized against beta-actin levels. The fold change in the level of each protein relative to pre-miR-negative-transfected control cells is shown on graph. Experiments were repeated twice and yielded smilar results. **(A)** Expression of Notch3 downstream molecules after pre-miR-150 transfection. **(B)** Expression of proteins of cell survival and cell cycle after pre-miR-150 transfection. **(C)** Expression of apoptosis related proteins after pre-miR-150 transfection. Significantly downregulated anti-apoptotic proteins were BCL-W (0.51-fold) and BCL-2(0.42-fold) and significantly upregulated pro-apoptotic protein was Bak (4.57-fold).

The expression of Notch3 downstream target proteins, including NICD3 and Hey2 were downregulated to a greater extent by pre-miR-150 transfection. At 24h, levels of NICD3 and Hey2 were significantly reduced to 0.56- and 0.67-fold, respectively, relative to control (*P*=0.05 and *P*<0.05, respectively). However, Hes1 and Hey1 tend to be reduced but not significantly downregulated by pre-miR-150 (Figure 5A). These results demonstrated that miR-150 might influence the expression of Notch3 downstream proteins.

We then analyzed the expression of proteins related to cell survival and cell cycle, including DNA-PK, pS6, S6, NF-κB, p21 and p27 in SKpac-13 cells treated with pre-miR-150. At 24h after transfection, expression levels of DNA-PK, pS6, S6, cyclin D3, p21, p27 and NF-κB were markedly decreased (DNA-PK: 0.18-fold; PS6: 0.25-fold; S6: 0.27-fold relative to control) and those of cell cycle proteins were also downregulated (cyclin D3: 0.36-fold; p21: 0.4-fold; p27: 0.08-fold; NF-κB: 0.10-fold relative to control) (*P<0.05) (Figure 5B).

As for the proteins related with apoptosis, treatment with pre-miR-150 significantly decreased the levels of the anti-apoptotic proteins, BCL-W, BCL-2, and BCL-XL by 0.51-fold, 0.42-fold, and 0.51-fold, respectively, relative to control (*P*=0.002, *P*=0.018, and *P*=0.094, respectively) (Figure 5C). The levels of five pro-apoptotic proteins, Bad, Bak, Bim, Bid and Bax were increased by 3.71-fold, 4.57-fold, 2.83-fold, 1.41-fold, and 1.23-fold, respectively, relative to control (*P*=0.375, *P*<0.0001, *P*=0.114, *P*=0.694, and *P*=0.322, respectively) (Figure 5C). These results demonstrate that miR-150 may induce apoptosis by inhibition of anti-apoptotic genes and increase of apoptotic genes in the PTX-resistant ovarian cancer cells.

## DISCUSSION

Despite high initial response rate to first-line platinum/taxane chemotherapy, most ovarian carcinomas relapse. Often diagnosed at an advanced stage, the high chemoresistance of ovarain HGSC has made it difficult to cure [1]. It is well known that resistance to chemotherapy is a major obstacle to long-term remission, and an effective strategy to overcome drug resistance is necessary. In the present study, we observed that miR-150 directly targets Notch3, an oncogene related with drug resistance in ovarian cancer, and that patients with HGSC were significantly associated with downregulation of miR-150, supporting that miR-150 is a tumor supperssor. We further demonstrated that experimental upregulation of miR-150 increased the apoptotic, anti-proliferative, and anti-migratory effects of PTX on PTX-resistant ovarian cancer cells.

In this study, qRT-PCR analysis showed that the expression level of miR-150 is significantly downregulated in ovarian HGSC tissues compared with benign serous tumor samples, suggesting that miR-150 acts as a tumor suppressor. According to the results regarding that PTX-resistant SKpac cell clones expressed significantly lower levels of miR-150 than the parental SKOV3 cells, our results implicate that downregulation of miR-150 correlated with the acquisition of taxane resistance in ovarian cancer cells. Our hypothesis was that artificial increase of miR-150 level induces sensitizing effect on miR-150 downregulated, PTX-resistant ovarian cancer cells, suggesting that induction of miR-150 may be a reasonable approach to improving drug sensitivity.

The miRNAs are potential diagnostic and prognostic markers in cancers including ovarian cancer [10, 15, 16]. Emerging evidence suggests that miRNAs affect to signaling molecules such as transforming growth factor-β, WNT, Notch and epidermal growth factor. As such, miRNAs serve as nodes of signaling networks that regulate cancer development, metastasis, and stem cell biology [17]. We herein report that miR-150 directly targets Notch3, which has been reported by many authors including us to be an oncogene related with chemoresistance and cancer stem cell activation in many human cancers [4, 5, 8]. Notch3 was overexpressed in more than 50% of ovarian HGSC tumors compared with the normal ovarian surface epithelium [6]. It has been published that inhibition of Notch3 signaling suppresses cell growth and induces apoptosis in breast [18], ovarian [19] and lung cancers [20]. While many authors reported that Notch3 plays a role in ovarian cancer cell proliferation, tumor growth, and metastasis, additional investigations revealed that Notch3 signaling is implicated in chemoresistance [7, 8]. Our previous studies also demonstrated that Notch3 overexpression was induced by long-term use of PTX and acquisition of PTX-resistance in ovarian cancer cells [4]. Therefore, miR-150 targeting Notch3 might be associated with chemoresistance as well as cancer development.

Recent studies have indicated that the aberrant expression of miR-150 is closely associated with tumorigenesis, cancer development, and a curative effect by influencing oncogenes and tumor suppressor genes [12, 21–25]. In cutaneous T-cell lymphoma, upregulation of miR-150 inhibited tumor invasion and metastasis by targeting the chemokine CCL20 receptor, CCR6 [21]. Lu *et al*. recently showed that miR-150 is an important regulator of hematopoietic recovery upon 5-fluorouracil (5-FU)-induced injury through targeting *Myb* [26]. The downregulation of miR-150 was related to platinum resistance in bladder tumor [24], however, the function of miR-150 in the development or regulation of chemoresistance in ovarian cancer has not been reported.

In the present study, we first report that miR-150 is related with PTX-resistance as well as functions as a tumor suppressor in ovarian HGSCs. We further focused on elucidating the impact of administration of pre-miR-150 on sensitizing the chemoresistant cancer cells, particularly those resistant to PTX. Results of WST, colony forming and TUNEL assays showed that pre-miR-150 treatment significantly decreased cell proliferation, and increased apoptosis in PTX-resistant SKpac cells. These results were amplified when co-treated with PTX. In this study, we observed 3-fold increase in apoptosis by pre-miR-150 in combination with PTX compared with that by pre-miR-150 alone, whereas both treatments showed similar reduction in clonal growth of SKpac cells by colony forming assay. It is very hard to explain the reason of its different effects on apoptosis and proliferation, but we speculate that pre-miR-150 alone can reduce the proliferation and induce the apoptosis in PTX-resistant ovarian cancer cells. In case of combined treatment of pre-miR-150 and PTX, pre-miR-150 resensitizes PTX-resistant cells to PTX, resulting in additive effect of pre-miR-150 and PTX on apoptosis, whereas additive effect does not occur on cell proliferation. The further study is needed to investigate this phenomenon.

In light of our previous report that Notch3 overexpression correlated with distant metastasis in HGSC [4], and that angiogenesis and migration are well known important factors governing tumor progression and metastasis, it is suggested that Notch signaling pathway may be involved in these processes. Liu *et al* [27] reported that Notch3 is an important regulator of pathological blood vessel formation, thus Notch3 knockdown may play a critical role in reducing angiogenesis, which was reported in our previous study [5]. In addition, Roca *et al* [28] suggested that the regulation of endothelial cell sprouting and proliferation are mediated by Notch3 pathway, suggesting the possible involvement of miR-150 in tumor angiogenesis. In this study, pre-miR-150 treatment showed inhibitory effects on cancer cell migration and tube formation (angiogenesis) in PTX-resistant SKpac cells, and this effect was not seen in PTX-treatment alone. Taken together, the restoration of miR-150 induces the drug-resistant ovarian cancer cells to regain the sensitivity to PTX, and inhibits cancer cell migration and proliferation, at least partly by the repression of its target gene, Notch3, and its downstream proteins.

Given that miR-150 regulates Notch pathway, and that inhibition of the Notch pathway can regulate tumor-initiating CSC populations, which are responsible for chemoresistance and cancer recurrence in many human cancers, we further determined whether pre-miR-150 treatment is able to suppress stem-like cell properties in SKpac cells. In accordance with our hypothesis, we found that PTX with pre-miR-150 transfection resulted in suppression of sphere forming activity, which represents the self-renewal activity of CSCs, whereas PTX alone brought about no change in spheroid formation. This result suggests that gain-of function of miR-150 enhances the chemosensitivity of ovarian cancer by targeting the cancer stem-like cell population.

To elucidate the mechanisms underlying the effects of Notch3 downstream proteins after pre-miR-150 treatment, we measured Notch3 intracellular domain (NICD3), Hes1, Hey1, and Hey2 expression levels in PTX-resistant SKpac cells. The NICD3 is required for activation of Notch3 target genes such as HES and HEY families, cyclin D1 (CCND1) and c-MYC that ultimately results in activation of cell cycle and inhibition of apoptosis. Overexpression of NICD3 was also characteristically observed in both cisplatin/paclitaxel-resistant ovarian cancer cells [28]. As expected, the expression levels of Notch3 downstream proteins, including NICD3, Hes1, Hey1, and Hey2, were downregulated in cells treated with pre-miR-150 compared to control cells treated with pre-miR-negative, and the results of both NICD3 and Hey2 were statistically significant. These results indicate that the effect of miR-150 functions on cancer cells at least partly by repressing Notch3 signaling pathway.

We also demonstrated that the expression of proteins related to cell proliferation and survival, including DNA-PK, pS6, S6, cyclin D3, p21, p27, and NF-κB, were reduced after pre-miR-150 transfection on PTX-resistant SKpac cells. As expected, the downregulation of cell cycle proteins was observed early, and the cell cycle inhibitory proteins (p21, p27) were initially downregulated and then upregulated after 48h. Although Notch3 is just one of the many targets of miR-150, we reasoned that reduced expression of Notch3 by pre-miR-150 might make the cells release the suppressive controller, such as p21 or p27, to allow cell cycle regulation.

To elucidate the mechanisms of apoptotic effect of miR-150, we observed apoptosis-related proteins after pre-miR-150 treatment. After transfection (24h), while anti-apoptotic proteins (BCL-W, BCL-2, and BCL-XL) tended to decrease, pro-apoptotic proteins (Bad, Bak, Bim, Bid and Bax) tended to increase. Among these genes, BCL-W, BCL-2, and Bak were significantly changed, suggesting that these genes are mainly involved in the regulation of apoptosis regulated by miR-150.

In the current study, we found that miR-150 targets *Notch3*, and that the artificial overexpression of miR-150 enhanced PTX sensitivity in PTX-resistant ovarian cancer cells by inhibiting proliferation and promoting apoptosis. Through a comprehensive examination, we revealed that forced expression of miR-150 altered expression of proteins related to cell survival, cell cycle, and apoptosis. We, therefore, provide novel evidence that modulation of the miR-150-*Notch3* axis is a promising therapeutic strategy for overcoming PTX-resistant ovarian cancer.

## MATERIALS AND METHODS

### Patients and tissue samples

Samples from 58 patients with high-grade ovarian serous carcinoma (HGSC) were obtained from the archives of the Department of Pathology, CHA Bundang Medical Center. The tissues were fresh snap frozen for quantitative RT-PCR. As a control, 4 cases of ovarian benign serous tumor samples were used. Written informed consents were obtained from all patients prior to surgery and this study was approved by the Ethical Committee of the CHA Bundang Medical Center.

### Ovarian carcinoma cell lines

The human ovarian serous adenocarcinoma cell line (SKOV3) was obtained from the American Tissue Type Collection (Manassas, VA, USA). Establishment of several paclitaxel (PTX)-resistant sublines (SKpac-10, 12, 13, 16, and 17) has been described previously [5]. Briefly, SKpac cells were established by continuous exposure of SKOV3 cells to a stepwise escalating concentration of PTX for more than 8 months. These cell lines were maintained in McCoy's 5A medium (Gibco/Life Technologies, Grand Island, NY, USA) supplemented with 10% fetal bovine serum, 100U/ml penicillin, and 10 μg/ml streptomycin. Cultures were incubated at 37°C in a humidified atmosphere containing 5% CO_2_.

### Transfection of miRNA mimics

Pre-miR-150 and pre-miR-negative were purchased from Ambion (Life Technologies, Carlsbad, CA, USA) and were transfected with Lipofectamine 3000 (Invitrogen/Life Technologies Carlsbad, CA, USA) reagent according to the manufacturer's instructions.

### Quantitative real-time PCR

Total RNA was extracted from fresh tissues and cell lines using TRIzol reagent (Invitrogen/Life Technologies Carlsbad, CA, USA) and reverse transcribed using specific miRNA primers and reagents from the TaqMan MicroRNA Reverse Transcription Kit (Applied Biosystems, Foster City, CA, USA). qRT-PCR for mature miRNAs was conducted using a Bio-Rad CFX96 Real-Time PCR Detection System (Bio-Rad, Hercules, CA). All PCR reactions were run in triplicate and gene expression relative to RNU48 was calculated using the comparative threshold method (2^−ΔΔCt^).

### Luciferase reporter assay

The 3′UTR segments of *Notch3* (human NOTCH3, NM_000435: chromosome 19:15270445-15271472;1028bp) were amplified by PCR from genomic DNA of SKpac-13, 16, 17 cells. The putative binding sites for miR-150 within the 3′UTR of *Notch3* were identified using the TargetScan algorithm (targetscan.org). The wild-type or mutated binding sites were cloned separately into the Nhel and Xhol sites of the pGL3-control vector (Promega, Mannheim, Germany). The pGL3-control (100 ng) and pRL-TK plasmids (5 ng; for normalization) were transfected into SKpac cells seeded in 24-well plates (3×10^4^ cells/well). Synthetic pre-miR-150 (Ambion/Life Technologies, Carlsbad, CA, USA) of 50 pmol were added to the above reactions. Luciferase activity was measured after 48 h on an Infinite 200pro series luminometer (Tecan Group, Zurich, Switzerland) using the Dual-Luciferase reporter assay system (Promega, Mannheim, Germany) according to the manufacturer's instructions. All experiments were performed in triplicate and normalized to Renilla luciferase activity.

### Spheroid-forming assay

Spheroid formation assays were performed to verify the effect of miR-150 on cancer stem cells activation. SKpac-17 cells were transfected with pre-miR-150 and pre-miR-negative was added. Cells were plated at a density of 1000 cells/cm^2^ in Ultra-Low Attachment Surface 6-well culture plates (Corning, Acton, MA, USA) in serum-free DMEM/F12 medium (Gibco/Life Technologies, Grand Island, NY, USA) supplemented with 20 ng/ml epidermal growth factor (Gibco, Carlsbad, CA, USA), 10 ng/ml basic fibroblast growth factor (Sigma–Aldrich, St. Louis, MO, USA), 0.4% bovine serum albumin (Sigma–Aldrich, St. Louis, MO, USA), and 5 μg/mL insulin (Sigma–Aldrich, St. Louis, MO, USA). Spheroid formation (50–100 cells per sphere) was assessed 7 days after seeding.

### WST assay

SKpac-12, 17 cells were seeded at 1 × 10^5^ cells/well in 6-well plates. The next day, cells were transfected with pre-miR-150 and incubated for 48 h. Transfected cells were then replated at 1 × 10^4^/well cells in a 96-well culture plate. After 48h, cells were measured by water-soluble tetrazoliumsalt (WST) assay according to the protocol of the Cell Counting Kit-8 (CCK8) assay kit (Dojindo, Japan). The absorbance was read at 450 nm using a microplate reader. All WST experiments were performed in triplicate and repeated at least three times.

### Colony-forming assays

SKpac-16, 17 cells were seeded at 1 × 10^5^ cells per well in six-well plates. On the next day, cells were treated with pre-miR-150 and incubated for 48 h. Treated cells were then replated at 300 cells per well in a six-well culture dish. After 14 days, colonies were visualized using hematoxylin after fixed with 4% paraformaldehide for 10 min and then counted colonies containing more than 50 individual cells.

### TUNEL assay for apoptosis analysis

TUNEL assays were performed after pre-miR-150 transfection in SKpac-12, and −17 cells. After 48 h, apoptotic cells were analysed using the *In Situ* Cell Death Detection kit (Roche, Mannheim, Germany). 2 × 10^7^ cells were fixed with 75% ethanol for 2 h at −20°C. They were washed twice with PBS and incubated in 0.1% Triton X-100 and 0.1% sodium citrate for 2 min on ice. After washing twice with PBS, the cells were incubated with a TUNEL labelling mixture for 1 h at 37°C in the dark. The samples were washed twice with PBS and analyzed by fluorescence activated cell sorting (FACS) (Becton Dickinson, Franklin Lakes, NJ, USA).

### Wound healing assay

Wound healing assays were performed after pre-miR-150 transfection in SKpac-12, and −17 cells. Cells were seeded into 24-well tissue culture plates and grown to confluency. An acellular area was created by scraping the cell surface using a sterile yellow pipette tip. The wounded monolayer was washed twice with PBS to remove floating cell debris. The monolayer was then incubated in cell culture medium and the rate of defect closure was monitored for 16 h. Individual cells were quantified as an average of at least five fields for each experiment.

### Angiogenesis assay

Matrigel (BD Biosciences, California, USA) was pipetted into flat-bottom, 96-well plate and polymerized for 1h at 37°C. HUVECs incubated in M199 containing 1% FBS for 5 h were harvested after trypsin treatment, resuspended in M199, plated onto the layer of Matrigel at a density of 1×10^4^ cells/well, and the supernatant of SKpac-17 cells transfected by pre-miR150 was added. After 24h, 48h, 72h, tube formation was observed.

### Western blot

Cells were lysed by protein extraction buffer (Pro-Prep, iNtRON Biotechnology, Korea) on ice for 30 min. After centrifugation at 4°C, 13,000 rpm for 15 min, the protein was measured with the Bradford assay (Sigma, Saint Louis, USA) from the supernatant. Same amounts of total protein were separated by 10% SDS-PAGE and transferred to nitrocellulose membranes (Millipore Co., Bedford, MA, USA). After blocked with 5% skim milk for 1 hr at room temperature, membranes were incubated overnight at 4°C in primary antibody (Table 1), followed by HRP conjugated anti-mouse 1:5,000 or anti-rabbit secondary antibody 1:5,000 (Novus Biologicals, Littleton, CO, USA) incubation for 1 hr at room temperature. Next, the bands of membranes were visualized by an ECL (enhanced luminol-based chemiluminescence) detection kit (Bio-Rad Laboratories, Hercules, CA, USA). The quantification of protein was done by densitometric digital analysis of protein bands using the ChemiDoc™ XRS+ with Image Lab™ Software (Bio-Rad Laboratories, Hercules, CA, US). Equal loading was confirmed by reprobing the membrane with beta-actin.

**Table 1:** List of primary antibodis used in western blotting

Antibody name	Company	Dilution
Anti-HES1	Novus Biologicals, Littleton, CO, USA	1:1000
Anti-HEY1	Proteintech, Chicago, IL, USA	1:1000
Anti-HEY2	Proteintech, Chicago, IL, USA	1:1000
Anti-DNA-PK	Epitomics, California, USA	1:1000
Anti-pS6	Epitomics, California, USA	1:10000
Anti-S6	Cell Signaling, Danvers, MA,USA	1:10000
Anti-Cyclin D3	Cell Signaling, Danvers, MA,USA	1:1000
Anti-NF-kB	Cell Signaling, Danvers, MA,USA	1:5000
Anti-p21	Epitomics, California, USA	1:500
Anti-p27	Cell Signaling, Danvers, MA,USA	1:1000
Anti-BCL-W	Epitomics, California, USA	1:500
Anti-BCL-2	Lab Vision, Fremont, CA, USA,	1:1000
Anti-BCL-XL	Epitomics, California, USA	1:1000
Anti-Bad	Epitomics, California, USA	1:1000
Anti-Bak	Cell signaling, Danvers, MA,USA	1:1000
Anti-Bim	Cell signaling, Danvers, MA,USA	1:1000
Anti-Bid	Epitomics, California, USA	1:1000
Anti-Bax	Cell Signaling, Danvers, MA,USA	1:1000
Anti-B-actin	Santa Cruz Biotechnology, Santa Cruz, CA, USA	1:10000

### Statistical analysis

Statistical analyses were performed using SPSS Software version V20.0.0 (IBM SPSS). The Student's t-test was used to determine the statistical significance between chemoresistant cell lines and controls. For each result, significant changes within the 95% confidence interval (*P*<0.05) were analyzed.
